# Change in the prevalence asthma, rhinitis and respiratory symptom over a 20 year period: associations to year of birth, life style and sleep related symptoms

**DOI:** 10.1186/s12890-018-0690-9

**Published:** 2018-09-12

**Authors:** Christer Janson, Ane Johannessen, Karl Franklin, Cecilie Svanes, Linus Schiöler, Andrei Malinovschi, Thorarinn Gislason, Bryndis Benediktsdottir, Vivi Schlünssen, Rain Jõgi, Deborah Jarvis, Eva Lindberg

**Affiliations:** 1 Department of Medical Sciences, Respiratory, Allergy and Sleep Medicine, Uppsala University, Uppsala, Sweden; 2 Centre for Clinical Research, Haukeland University Hospital, Bergen, Norway; 3 Dept. of Surgical and Perioperative Sciences, Surgery, Umea University, Umea, Sweden; 4 Institute of Clinical Science, University of Bergen, Bergen, Norway; 5 Department of Occupational and Environmental Medicine, Sahlgrenska University Hospital, Gothenburg, Sweden; 6 Department of Medical Sciences, Clinical Physiology, Uppsala University, Uppsala, Sweden; 7 Department of Respiratory Medicine and Sleep, the National University Hospital of Iceland, Reykjavik, Iceland; 8 Faculty of Medicine, University of Iceland, Reykjavik, Iceland; 9 Department of Public Health, Section for Environment, Occupation and Health, Aarhus University, Aarhus, Denmark; 10 National Research Center for the Working Environment, Copenhagen, Denmark; 11 Lung Clinic, Tartu University Clinics, Tartu, Estonia; 12 Respiratory Epidemiology, Occupational Medicine and Public Health, National Heart and Lung Institute, Imperial College, London, UK

**Keywords:** Asthma, Allergic rhinitis, Obesity, Smoking, Gastroesophageal reflux

## Abstract

**Background:**

The aim of this investigation was to study change in adults over a 20 year period in the prevalence of respiratory symptoms and disorders and its association to year of birth, life style and sleep related variables.

**Method:**

Adults 20–44 years of age, 6085 women and 5184 men, were randomly selected from seven centres in Northern Europe and followed for 20 years. The number of participants in the first survey was 21,595 and 11,269 participated in all three surveys. The participants were divided into three birth cohorts: 1944–1955, 1956–1965 and 1966–1975.

**Results:**

During the 20 year period the prevalence of wheeze decreased (− 2%) and the prevalence of asthma (+ 4%) and allergic rhinitis (+ 5%) increased, whereas the prevalence of nocturnal respiratory symptoms was relatively unchanged. The increase in allergic rhinitis was largest in those born 1966 to 1975 except in Estonia. There was large decrease in smoking (− 20%), increase in obesity (+ 7%) and snoring (+ 6%) during the study period. Smoking, obesity, snoring and nocturnal gastroesophageal reflux (nGER) were related to a higher risk of all symptoms. Obesity, snoring and nGER were also independently related to asthma.

**Conclusion:**

We conclude that as our participants got older there was a decrease in wheeze, no change in nocturnal symptoms and an increase in reported asthma and allergic rhinitis. These changes in prevalence are probably related to a decrease in smoking being counteracted by an increase in allergy, obesity and sleep related disorders.

## Background

Studies investigating time trends in asthma and respiratory symptoms by repeated cross sectional analyses have shown a decrease in the prevalence of some respiratory symptoms, a moderate increase of self-reported asthma and a sharp increase in the prevalence of allergic rhinitis in the last two decades [1–3]. There is, however, limited information available on how the prevalence of respiratory symptoms, asthma and allergic rhinitis changes with age. In the follow up of the European Community Respiratory Health Survey (ECRHS II) 11,000 young adults were followed for 10 years [4]. No large change in respiratory symptoms was found with age, but the prevalence of self-reported asthma and allergic rhinitis increased. The greater change in prevalence of allergic rhinitis was found in the youngest age group. In the second follow-up (ECRHS III), we see a decrease in the prevalence of wheeze while the prevalence of reported asthma continued to increase [5]. Other longitudinal studies of adults have shown diverging results with an increase in wheeze and cough with age in an English and a Canadian study [6, 7] and a decrease in the prevalence of wheeze in a German study [8].

Subsequent analyses of data from ECRHS II has shown an increase in obesity [9] and a sharp decrease in smoking [10]. Obesity was a strong risk factor for onset of respiratory symptoms in the Respiratory Health In Northern Europe study (RHINE) which is a 10 year follow-up of participants in the ECRHS from the Nordic countries [11]. It is possible that lack of change in the prevalence of respiratory symptoms with age in the ECRHS II [4], was related to the decrease in smoking and increase in obesity counteracting each other. In the RHINE study we also found that onset of respiratory symptoms and self-reported asthma was related to sleep related variables such as snoring and nocturnal gastroesophageal reflux (nGER) [11].

In 2010–2012 we conducted a second follow-up of the RHINE population (RHINE III) [12]. This follow-up also included information on body mass index (BMI), smoking, snoring and nGER. It is therefore now possible to investigate change in respiratory health by age and factors related to this change over a 20 year period. The aim of this investigation was to study change in the prevalence of respiratory symptoms and its association to year of birth, smoking, obesity, snoring and nGER.

## Methods

ECRHS stage I took place in 1990–1994. In stage I of the ECRHS, males and females aged 20–44 years were randomly selected from the population register in the participating centres [13]. A postal questionnaire was sent to 3000–4000 subjects at each centre. From those who responded, a random sample was selected to undergo a more detailed clinical examination (stage 2). In addition a “symptomatic sample”, reporting symptoms of waking with shortness of breath, asthma attacks or using asthma medication in stage 1 were also studied. The clinical examination included a structured interview where the subjects were asked about symptoms, respiratory disorders, smoking and other types of exposure [13]. The examination also included allergy testing, spirometry and measuring height and weight. In some centres an additional questionnaire collecting data on sleep disturbances was used [14].

RHINE II is a follow-up study of participants from ECRHS stage 1 from seven Northern European centres: Reykjavik (Iceland); Bergen (Norway); Aarhus (Denmark); Gothenburg, Uppsala, Umea (Sweden); and Tartu (Estonia) [11]. RHINE II consisted of a postal questionnaire sent in 1999–2001. In 2010–2012 a second follow-up by postal questionnaire was performed in the same centres (RHINE III) [12, 15]. The questionnaire was once again sent out to all participants of ECRHS stage 1. A summary of the number of participant and the data collected is presented in Table 1.

**Table 1:** Number of participants with available data

	Respiratory symptoms	Smoking	BMI	Sleep related
1990–1994	21,595	14,780	6604	1867
1999–2001	16,049	15,930	15,930	15,762
2010–2012	13,093	12,738	12,930	12,811
≥ 2 surveys	17,711	13,117	12,504	11,338
All three surveys	11,269	10,858	3669	1143

Informed consent was obtained from each participant and the study was approved by regional committees of medical research ethics in each country.

### Respiratory health

In all three surveys identical yes/no-questions were posed about presence of respiratory symptoms at any time in the last 12 months: wheezing, nocturnal chest tightness, nocturnal shortness of breath and nocturnal cough. Subjects were considered to have asthma if they reported that they currently were using medication against asthma or have had an attack of asthma with the last 12 months [16]. Allergic rhinitis was defined as a positive answer to the question “Do you have any nasal allergies including allergic rhinitis?”. In some of the analyses nocturnal chest tightness and nocturnal shortness of breath were combined and labeled nocturnal dyspnea [11].

### Year of birth

The participants were divided into three birth cohorts: 1944–1955, 1956–1965 and 1966–1975.

### Life style factors

#### Smoking

Questions on smoking were not included in the first survey in all centres. The second and third survey did, however, include questions on ex- and current smoking as well as age of starting and stopping to smoke. This information was used to estimate whether or not a participant was a smoker at the first survey or not. The question: “Are you a smoker?” was used to define current smokers in the second and third survey.

#### Body mass index

Body mass index (BMI) was calculated for each subject as weight in kilograms divided by the squared height in meters (kg/m^2^). Subjects with BMI ≥30 were classified as being obese. Information on height and weight was only collected from one centre in the first postal survey (Bergen). In the other centres data on height and weight collected on the subsample that underwent the clinical investigation (stage 2) was used for the first time period. In the second and third survey information on height and weight were collected from questionnaires in the same way in all centres.

### Sleep related variables

The postal questionnaire in survey two and three contained several multiple-choice questions in which the subjects were asked to estimate the frequency of various sleep related symptoms on a 5-points scale: never; less than once a week; one to two nights a week; three to five nights a week and almost every night [11]. For the first survey similar information was only available from the subsample that underwent the clinical examination in three of the centres: Reykjavik, Gothenburg and Uppsala.

The question asked regarding nocturnal gastroesophageal reflux was: “Do you have heartburn or belching when you have gone to bed?” Subjects reporting these symptoms 1 to 2 nights per week or more, are in this study referred to as reporting nGER [11]. The question asked regarding snoring was: “Do you snore loudly and disturbingly?” Subjects reporting such snoring 3 to 5 times per week or more are referred to as reporting habitual snoring [17].

### Statistics

Absolute net change in symptom and disease status between the surveys was estimated using population averaged, generalised estimating equations for a binomial outcome with identity link, with participants identified as the clustering factor and the number of the survey as an independent variable. Results were expressed as net between the surveys. The Wald test was used to examine differences in change of prevalence by birth cohort.

The influence of year of birth, life style (smoking and BMI)and sleep related variables (snoring and nGER) on respiratory health was analysed using mixed effects logistic regression in order to take into account that number of times information on risk factors was available varied between the participants.

Estimates of the influence of birth year on allergic rhinitis by centre were examined for heterogeneity and combined using random effects meta-analysis.

## Results

**Fig. 1:**
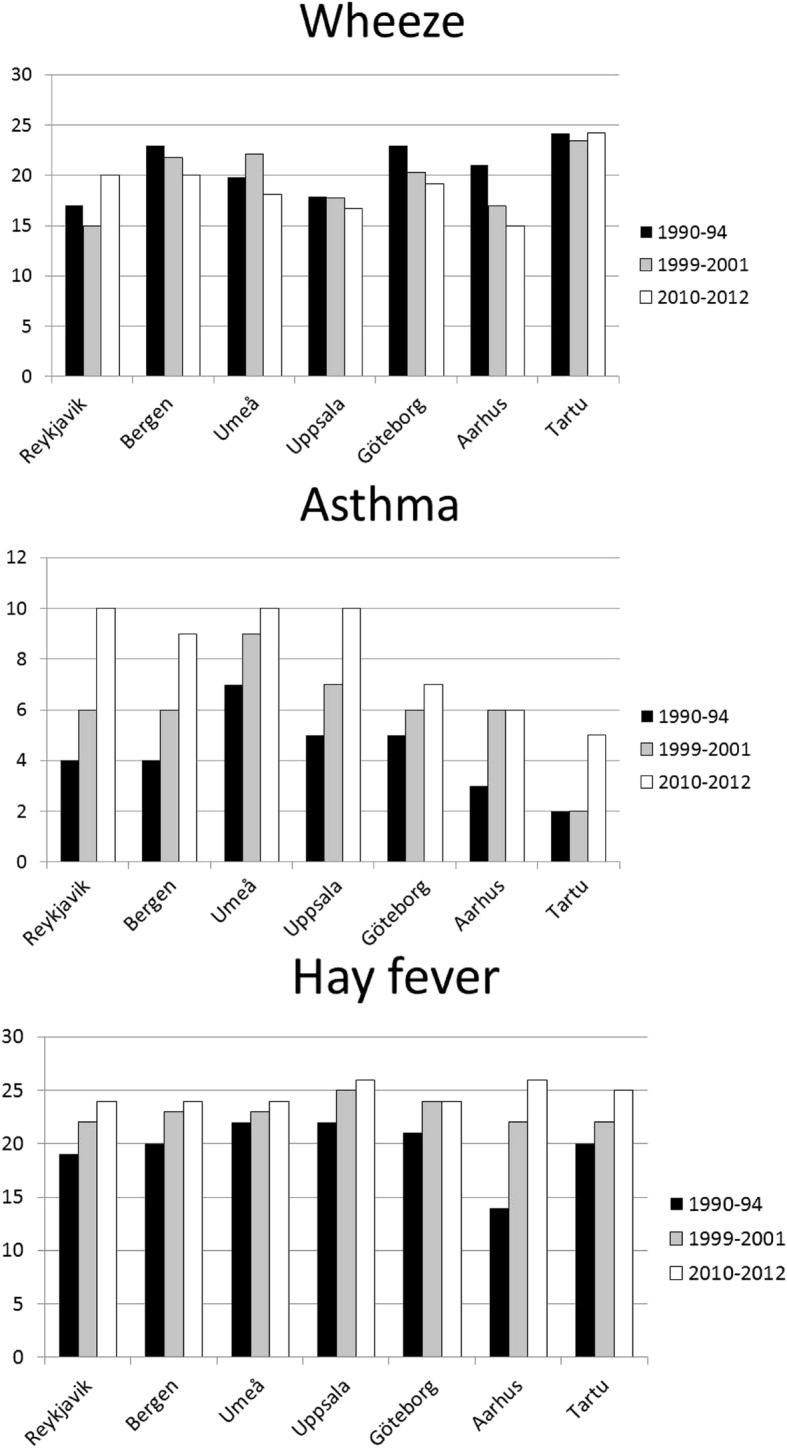
Prevalence of symptoms for subjects who participated in all three surveys (%) divided by centre

The prevalence of respiratory symptoms, asthma and allergic rhinitis for those 11,269 subjects (6085 women and 5184 men, mean age (±SD) in first survey 32.2 ± 7.1 years) that participated in all three studies is presented in Table 2. There was a clear decrease in the prevalence of wheeze and an increase in the prevalence of asthma and allergic rhinitis, whereas the prevalence of nocturnal respiratory symptoms was relatively unchanged. Change in the prevalence of wheeze, asthma and allergic rhinitis by centre is presented in fig. 1. Overall the trends were the same in all centres except for wheeze where the prevalence did not decrease in two of the seven centres (Reykjavik and Tartu). There was a large decrease in the prevalence of smoking while an increase in obesity and snoring was found when comparing the second and third survey where data was available for a larger proportion of the population (Table 2).

**Table 2:** Prevalence of symptoms, disorders and lifestyle and sleep related variables for subjects who participated in all three surveys (%, *n* = 11,269)

	Prevalence %			Change in prevalence % (95% CI)	
	1990–94	1999–2001	2010–2012	1990–2012	*p*-value
Wheeze	20.4	19.5	18.6	−2.2 (−3.0, −1.5)	< 0.0001
Nocturnal chest tightness	11.1	11.3	10.3	−0.6 (−1.2, 0.1)	0.08
Nocturnal breathlessness	4.7	4.6	5.3	0.4 (−0.1, 0.8)	0.10
Nocturnal dyspnea	11.1	11.3	10.6	−0.7 (−1.2, − 0.01)	0.02
Nocturnal cough	26.6	28.2	26.9	0.2 (−0.6, 1.1)	0.55
Asthma attacks	2.8	3.3	3.5	0.9 (0.5, 1.2)	< 0.0001
Asthma medication	3.6	5.5	7.6	4.1 (3.7, 4.5)	< 0.0001
Asthma	4.5	6.4	8.3	4.0 (3.5, 4.5)	< 0.0001
Allergic rhinitis	19.7	23.1	24.7	5.1 (4.5, 5.8)	< 0.0001
Smoking	37.7	25.7	16.4	−19.6 (−20.2, −18,9)	< 0.0001
Obesity (BMI > 30 kg/m2)		7.9	14.9	6.9.5 (6.4, 7.5)	< 0.0001
Snoring		17.8	24.2	6.3 (5.6, 7.2)	< 0.0001
Gastroesophageal reflux		7.0	7.3	0.2 (−0.4, 0.6)	0.57

**Fig. 2:**
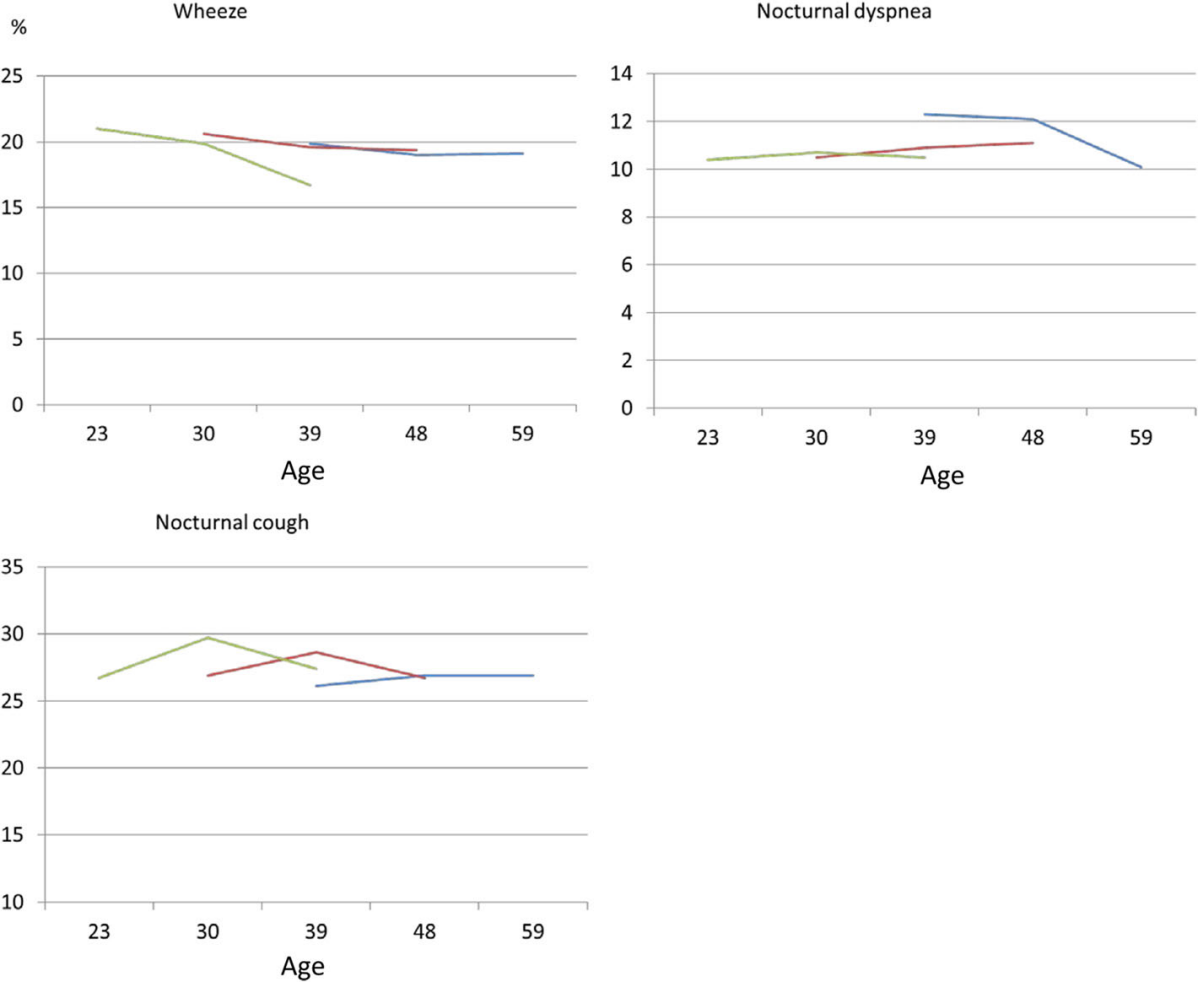
The prevalence of respiratory symptoms in relation to mean age at the three surveys for participants born 1945–1955 (blue), 1956–1965 (red) and 1966–1975 (green), respectively

**Fig. 3:**
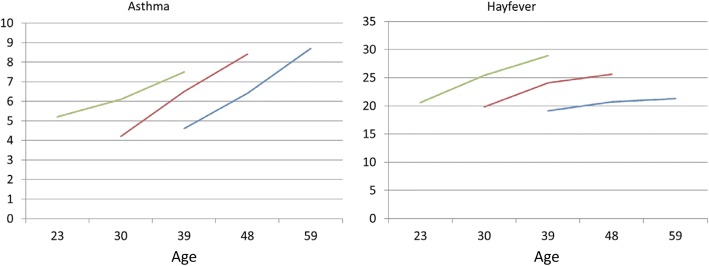
The prevalence of self-reported asthma and allergic rhinitis in relation to mean age at the three surveys for participants born 1945–1955 (blue), 1956–1965 (red) and 1966–1975 (green), respectively

The prevalence of symptoms, asthma and rhinitis in the three surveys relation to year of birth is presented in figs. 2 and 3. The youngest group (born 1966–1975) had the highest decrease in wheeze and nocturnal dyspnea, the lowest increase in asthma and the highest increase in allergic rhinitis.

Snoring and nGER were independently related to all respiratory symptoms, asthma and allergic rhinitis (Table 3). BMI was associated with all symptoms and asthma but not to allergic rhinitis. Smoking was related to a higher risk of all symptoms but not asthma and allergic rhinitis. Women had a much higher risk than men of nocturnal cough but also of all other symptoms and disorders except wheeze.

**Table 3:** Year of birth, gender, life style and sleep related variables in association with respiratory symptoms and disorder (adjusted^a^ odds ratio (95% CI))

	Wheeze	Nocturnal dyspnea	Nocturnal cough	Asthma	Allergic rhinitis
Born 1945–1955	1	1	1	1	1
1956–1965	1.04 (0.92–1.18)	0.93 (0.82–1.05)	1.03 (0.93–1.14)	1.03 (0.84–1.27)	1.62 (1.32–1.99)
1966–1975	1.14 (0.99–1.33)	0.95 (0.81–1.10)	1.11 (0.99–1.25)	1.05 (0.82–1.34)	2.36 (1.85–3.01)
Women	1.09 (0.97–1.22)	1.26 (1.13–1.41)	2.35 (2.14–2.57)	1.66 (1.38–2.01)	1.58 (1.32–1.89)
Smokers	5.65 (5.00–6.38)	1.68 (1.49–1.89)	2.04 (1.86–2.25)	0.98 (0.81–1.18)	0.60 (0.51–0.72)
Obesity	2.97 (2.55–3.45)	1.69 (1.45–1.96)	1.78 (1.57–2.02)	2.05 (1.62–2.60)	0.86 (0.68–1.09)
Snoring	1.85 (1.64–2.09)	1.71 (1.51–1.94)	1.56 (1.41–1.72)	1.61 (1.33–1.95)	1.42 (1.19–1.70)
Gastroesophageal reflux	2.85 (2.41–3.37)	3.67 (3.13–4.31)	2.50 (2.17–2.88)	2.78 (2.17–3.57)	1.73 (1.34–2.23)

^a^adjusted for centre and all the variables in the table

**Fig. 4:**
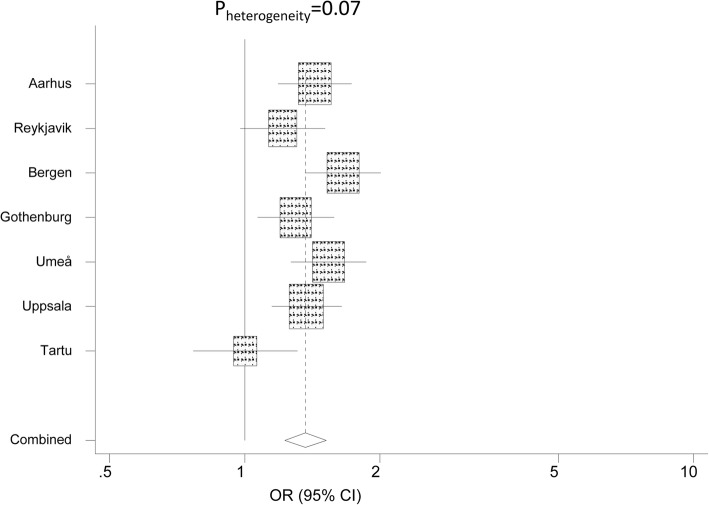
Odds ratio for allergic rhinitis in those born after 1965 compared to those born before 1956 by study centre. The odds ratio is adjusted for sex, smoking, body mass index, gastro-esophageal reflux and centre. The area of each square is proportional to the reciprocal of the variance of the estimate for the country. The combined random effects estimate is shown by the dashed line, the diamond having the width of its 95% confidence interval

The risk of allergic rhinitis was higher in those born 1966 or later than in those born before 1956 (Table 3). This association was also studied by centre (Fig. 4). A strong association between belonging to the younger age group and allergic rhinitis was seen in all centres except Tartu and a borderline centre heterogeneity was found for this association (*p* = 0.07).

## Discussion

The main result of this longitudinal study is that wheeze decrease, nocturnal respiratory symptoms remain unchanged and asthma and allergic rhinitis increases with age. The increase in allergic rhinitis was particularly strong in participants born after 1965 except in Estonia. Part of these changes in symptoms and disorders may be related to changes in life style (smoking, diet and exercise) and sleep related factors with a large decrease in smoking but an increase in obesity and snoring with age [9].

Our finding of a decrease in wheeze is in accordance with the results of analyses of repeated cross sectional studies in Northern Europe [1, 2]. An increase in self-reported asthma was found in the 10 and 20-year follow up of the ECRHS [4](ref) and in one Swedish repeated cross sectional studies [1], while an increase in allergic rhinitis has been found in a large number of studies [1–3, 18].

The increase in allergic rhinitis was largest in the youngest birth cohort – those born between 1966 and 1975. Analyses of risk factors also showed that belonging to the youngest birth cohort increased the likelihood of having allergic rhinitis more than two fold. This risk association was found in all centres except in Tartu in Estonia. This result fits well with data from the ECRHS showing a lower prevalence of atopy in the eastern part of Germany [19] and a corresponding difference between Estonia and Sweden [20]. In both these studies difference in atopy was larger in the younger than the older birth cohorts. Corresponding geographical differences have also been reported from a number of studies on children [21, 22]. The lack of age association with allergic rhinitis in Tartu is probably related to the fact that Estonia during the cold war period underwent less environmental changes than those that occurred in the Scandinavian countries.

Smoking and obesity were as expected associated with respiratory symptom [11, 23–26]. Obesity was also associated with self-reported asthma whereas this was not the case for smoking. During the follow up there was a large decrease in smoking and an increase in obesity. Other cohort studies. Have also found a clear positive association between incidence of wheeze and smoking [6, 7, 27]. We have previously found that smoking cessation and weight gain counteract each other when it comes to the effect of change in lung function [9]. It is therefore probably that the relative stability found in the prevalence of all respiratory symptoms except wheeze is related to the beneficial effect of less smoking being balanced by the negative effect of increasing obesity.

Snoring was as in previous studies found to be associated with wheeze and nocturnal symptoms [11, 28]. In accordance to previous studies we also found an association between nGER and respiratory symptoms and disorders [11, 29–31]. Snoring became more prevalent as our population got older. This may partly be related to weight gain and may together with increasing obesity explain why there was no change in nocturnal respiratory symptoms despite a large decrease in smoking.

The strengths of this study are the large population size and the long follow-up time. The questions used are standardized and have been used in a large number of previous studies [32, 33]. The response rate is acceptable given the long follow-up time and the long term responders were fairly similar to non-responders in term of symptomatology [12]. A draw-back is that our results are only based on self-reported data and that there was a variation in the amount of data that was available at the different surveys.

## Conclusion

We conclude that as our participants got older there was a decrease in wheeze, no change in nocturnal symptoms and an increase in reported asthma and allergic rhinitis. The increase in allergic rhinitis was particularly strong in younger adults except in Estonia. These changes in prevalence are probably related to a decrease in smoking being counteracted by an increase in allergy, obesity and sleep related disorders. Measures targeting obesity and sleep disordered breathing maybe important in order to reduce the burden of respiratory disorders in the society.

## Abbreviations

BMI: Body mass index
ECRHS: European Community Respiratory Health Survey
nGER: nocturnal gastroesophageal reflux
RHINE: Respiratory Health In Northern Europe study
